# Grafts of porcine small intestinal submucosa seeded with cultured homologous smooth muscle cells for bladder repair in dogs

**DOI:** 10.1186/1751-0147-55-39

**Published:** 2013-05-07

**Authors:** Victor JV Rossetto, Lígia SLS da Mota, Noeme S Rocha, Hélio A Miot, Fabrizio Grandi, Cláudia VS Brandão

**Affiliations:** 1 Department of Veterinary Surgery and Anesthesiology, School of Veterinary Medicine and Animal Science, São Paulo State University (UNESP), Rubião Júnior, S/N, Botucatu, ZIP COD 18.618-970, Brazil; 2 Department of Clinics, School of Veterinary Medicine and Animal Science, São Paulo State University (UNESP), Rubião Júnior, S/N, Botucatu, ZIP COD 18.618-970, Brazil; 3 Department of Dermatology and Radiotherapy, School of Medicine, São Paulo State University (UNESP), Rubião Júnior, S/N, Botucatu, ZIP COD 18.618-970, Brazil; 4 Department of Genetic, Institute of Biosciences, São Paulo State University (UNESP), Rubião Júnior, S/N, Botucatu, ZIP COD 18.618-970, Brazil

**Keywords:** Tissue reconstruction, Bioengineered tissue, Cell therapy, SIS

## Abstract

**Background:**

Due to numerous complications associated to gastrointestinal augmented cystoplasty, this study aimed to analyze the anatomic repair of the bladder of 10 female dogs using grafts of porcine small intestinal submucosa (SIS) seeded with cultured homologous smooth muscle cells, and compare them with the acellular SIS grafts.

**Results:**

We assessed the possible side effects and complications of each type of graft by clinical examination, abdominal ultrasound and laboratory findings. Anatomic repair of neoformed bladder was assessed by histological staining for H/E and Masson's Trichrome, analyzed with a Nikon Photomicroscope connected to the system of image analysis Image J.

**Conclusions:**

We propose that SIS associated to homologous smooth cells can improve the quality of tissue repair, and consequently decrease the potential complications inherent to acellular SIS.

## Background

The indications for surgical repair of the bladder include trauma with extensive tissue loss, recurrent interstitial cystitis, inflammatory pseudotumors, neurological dysfunctions and congenital genitourinary abnormalities [1-4]. In all these situations, bladder reconstruction, after partial cystectomy, has been traditionally performed by the transposition of autologous segments of the stomach or the intestine [1,5,6]. However, the cystoplasty using gastrointestinal segments may result in numerous infection, urolithiasis and retraction of the graft [3,4,7]. Due to these complications, many types of grafts have been used for bladder tissue repairing, alloplastic or biodegradable, with or without cell implantation. Among the different materials used, the porcine intestinal submucosa (SIS) is the most studied one and it stands out because of its capacity to repair the bladder and other tissues of the urinary tract [8].

The neoformed bladder from the SIS is microscopically similar to the normal one, but histologically different as regards the quantity and organization of the smooth muscle fibers, and presents in a disorganized way and in lower quantity in the bladders repaired with SIS [9]. An alternative to decrease these occurrences and to more accurately repair the anatomy of the bladder is the cell implantation associated to tissue bioengineering.

The aim of this work was to comparatively analyze the bladder anatomic reparation of dogs using acellular SIS seeded with bladder homologous smooth muscle cells (BSMC), and to evaluate the possible local and systemical complications, inherent to each type of applied graft.

## Methods

### Animal selection

We used 10 mixed-breed, female dogs from the School of Veterinary Medicine and Animal Science of Univ. Estadual Paulista (UNESP), campus of Botucatu. All the procedures were approved by the Ethics Committee of Animal Use. The animals were randomly distributed in two groups: Control Group (CG), constituted by five animals submitted to cystoplasty with acellular commercial SIS (Cook Biotech); and Treated Group (TG), constituted by five animals submitted to cystoplasty with graft of SIS, 3 cm wide × 4 cm long, seeded with homologous smooth muscle cells (HSMC).

### Cell culture

The bladder smooth muscle cells (BSMC) were obtained from bladder fragments from animals of the CG during the cystoplasty, and from other two mixed-breed dogs that died at the Veterinary Hospital of School of Veterinary Medicine and Animal Science/UNESP, campus of Botucatu.

The cells were cultivated in monolayer at the bottom of culture flasks filled with Ham F-12 culture medium, added with bovine fetal serum 20% and penicillin/streptomycin associated to Amphotericin B 1%, kept at the temperature of 36, 7°C and CO_2_ pressure of 5%. To confirm the cell type and validate the cultivation, an immunocytochemistry technique by the LSAB (Labeled StreptAvidin Biotin) method was performed. After an average of three passages and about 70% of cell confluence, they were seeded above the SIS under the same conditions described before.

In order to have the cells seeded into the SIS only, and to prevent the latter from floating on the cell culture, glass weights were placed onto each corner of the membrane, thus holding it to the bottom of the cell culture flasks.

### Surgical procedures

The resection of complete depth, 3 cm length X 4 cm width, at the ventral face of the bladder was performed to the cytoplasty of animals from both groups. We used the acellular SIS in the animals from the CG and SIS seeded with HSMC in the animals from the TG, sutured to the edges of the remaining bladder wall with polyglactin 910 3–0 suture. In the dogs from the TG, the surface of the seeded SIS was allocated at the extraluminal face of the bladder. The approximation or fixation of the omentum to the bladder was not performed. In order to accurately identify the grafted area, 3–0 nylon sutures were made along the original bladder and 0.5cm from the transition line between the graft and the original tissue.

Sixty days after the operation, all the dogs were submitted to a biopsy of complete depth of the bladder wall, in two distinct areas: a) central area of the cystoplasty; and b) transition area between the neoformed tissue and original adjacent bladder. At that moment, the microscopic evaluation of the inner and outer reparation of the bladder wall was performed.

### Histopathological and quantitative analysis of the muscle and collagen fibers

The samples were submitted to hispathological evaluation by H/E and Masson’s Trichrome stains. To quantify the collagen and muscle fibers, the histological laminas stained with Masson Trichrome were digitalized using the Nikon Coolscope II digital microscope. In both groups, a photomicrograph was performed from the central area and from the transition area between the neoformed tissue and the adjacent bladder, using 5 × objectives. Each image was processed by the ImageJ software using the K-means Clustering plugin. The number of clusters varied from five to seven, according to the micrograph contrast.

### Clinical, laboratorial and abdominal ultrasonographic evaluation

A postoperative evaluation was performed at 24 (M1) and 72 (M3) hours, and later on the 7^th^ (M7), 30^th^ (M30) and 60^th^ (M60) days. The complete blood count and the urine type I exam (urinalysis) were performed on days M7, M30 and M60, meanwhile the biochemical exams and urine type II (aerobic cultivation) were performed at M60.

All the animals were submitted to the abdominal ultrasonographic exam before the cystoplasty and at M30 and M60. During the evaluation period, no urinary derivation was performed.

### Statistical analysis

All the tests were performed using the IBM SPSS 20 software, considering p ≤ 0.05.

## Results

### Cell culture

The BSMC average initial counting of the animals from the CG was 1.5 × 10^5^ cells/mL, meanwhile the counting of donors 1 and 2 was 4.5 × 10^5^ and 6 × 10^5^ cells/mL, respectively. In both situations the cell viability was approximately of 95%.

The cells adhered to bottom of the flasks between the 1^st^ and 5^th^ days of cultivation. Immediately after their adherence, the BSMC started the mitosis process, assuming the flattened and elongated polygonal morphology, with cell projections and cytoplasmatic structures looking like contractible myofilaments. The number of cells increased exponentially until they achieved about 70% of cell confluence, when they were seeded above the SIS. The amount of seeded cells was from 7 × 10^5^ to 7.5 × 10^5^cells/mL

The immunocytochemistry technique using the LSAB method revealed a positive brownish marking for the alpha-actin soft muscle antibody.

### Macroscopic evaluation of grafted bladders

The reparation tissue at the outer face of the bladder of all the animals from the CG and TC, at M60, showed a glowing/shinning, plane and smooth surface, continuous to the original bladder tissue.

At the inner surface of the bladder, a reparation tissue was visible with a shinning surface in all the dogs from the TG and in two animals from the CG (p=0.17); in the other three animals from the CG, the inner surface was opaque.

In addition, at the cystoplasty location, a plane surface appeared in three animals from the CG and in all the animals from the TG; of smooth aspect in two animals from the CG and in three animals from the TG; and of irregular aspect in three dogs from the GC (two of them presenting a depression area in association) and in two animals from the TG. The continuity of the reparation tissue with the original tissue was considered adequate in two animals from CG and in all the animals from the GT, and inadequate in three animals from the GC (p=0.17).

In three animals from the GC uroliths of different sizes and shapes were observed, adhered to the inner surface of the SIS. Such findings were not observed in the animals from the TG (p<0.01).

### Histopathological and quantitative analysis of the collagen and muscle fibers

In the central samples of the animals from the CG, at the mucosal tunic, heterogeneous areas of urothelial covering were observed, from of one or two cell layers, to the complete absence of the urothelium. In the animals from the TG, little heterogeneous areas of urothelial covering were observed, with thick urothelium in their almost entirety.

**Figure 1:**
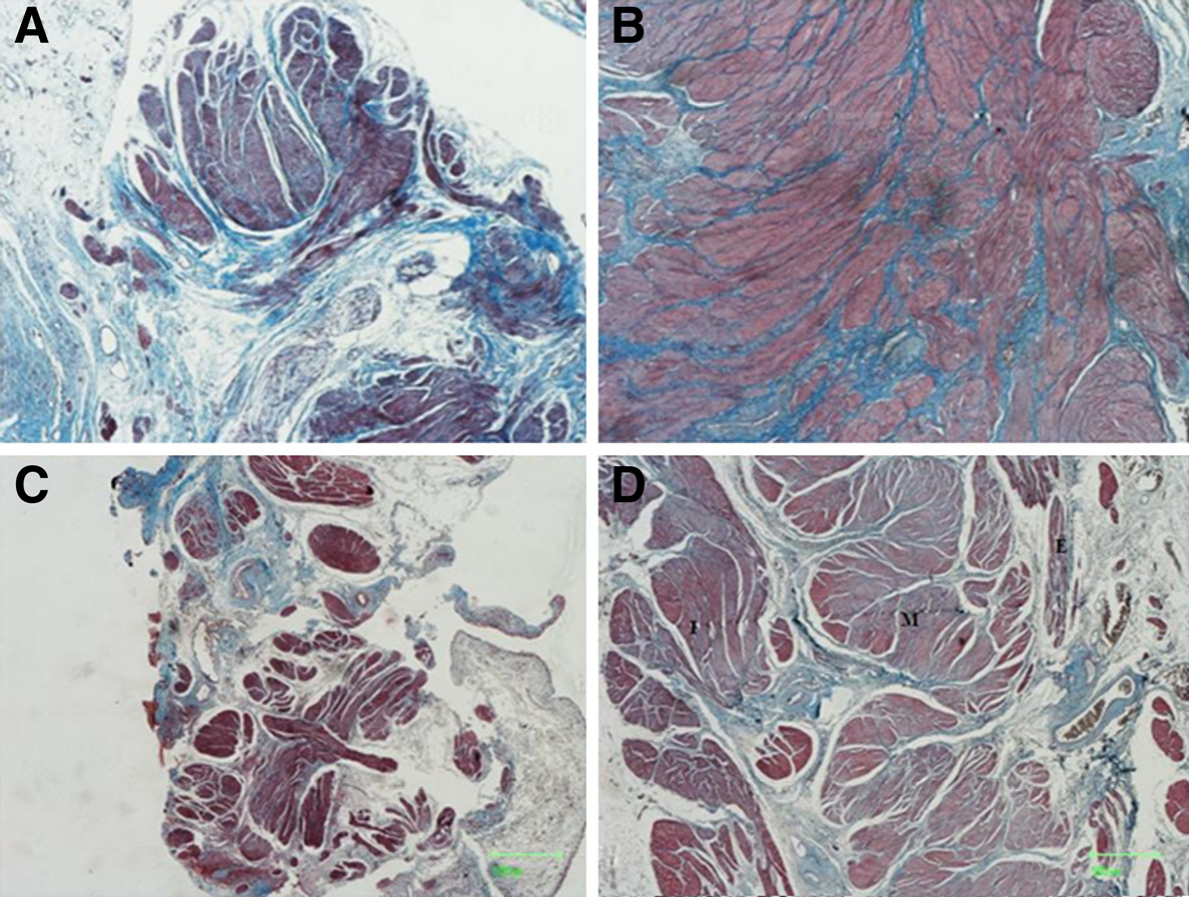
**Bladder lesions repaired with acellular and seeded SIS. A)** Photomicrograph of the central region of the bladder lesion repaired with the acellular SIS (CG). Note the absence of typical histological architecture and presence of disorganized muscle fibers colored in red, deposed in bundles of several gauges. (Masson, 4x). **B)** Photomicrograph of the central region of the cystoplasty repaired with the SIS seeded with HSMC (GT). Note the numerous muscle fascicle colored in red (Masson, 12,5x). **C)** Photomicrograph of the transition fragment between the acellular SIS and the original bladder (CG). Note the randomly arranged muscle fibers of different gauges (Masson, 2x). **D)** Photomicrograph of the fragment of the transition between the SIS seeded with HSMC and the original bladder (TG). Note the typical disposition of the outer longitudinal (E), medial circular (M), inner longitudinal (I) layers of the muscular tunic (Masson, 2x).

Still in the central samples of the animals from the GC, muscle fibers in disorganized bundles and different gauges were found in the muscular tunic. In addition, it was noted the absence of muscle tunic in three dogs (Figure 1A). In the animals from the TG, there was a typical organization and architecture, characterized by the presence of the outer longitudinal, circular medial and inner longitudinal layers (p=0.17) (Figure 1B). The thickness of the muscle tunic in the central area of the CG could not be measured. The average thickness of the muscle tunic in the central samples from the TG was 2679.55 μm.

Similarly, in the transitional samples of the animals from the CG, very heterogeneous areas of urothelial covering were observed in the mucosal tunic. In the animals from the TG, areas of homogenous urothelial covering were found, consisting of few cell layers in almost the entire length of the urothelium. Comparing both the groups, complete urothelic covering in the transition samples was superior on the TG (p=0.05), and urothelic hyperplasia was superior on the CG (p**<**0.01).

Still in the transition samples of the animals from the CG, muscle fibers of different gauges were observed, randomly distributed and without typical histological organization (Figure 1C). In the animals from the TG, there was typical organization and architecture, characterized by the presence of the outer longitudinal, circular medial and inner longitudinal layers (Figure 1D). The presence of typical stratification of the muscle tunic in the transition samples was higher in the TG than in the CG (p=0.05). The average thickness of the muscle tunic in the transition samples was 2809.86 μm in the TG and 3366.21 μm in the GC (p=0.42).

The main histological alterations, observed in the central and transition samples, are shown in Table 1. Table 2 and Table 3 show, respectively, the average percentage of the muscle and collagenous tissues on the central and transition areas of the dogs from the CG and TG.

**Table 1:** Major histological alterations observed in the central and transitional samples of animals from control (CG) and treated (TG) groups 60 day after the cystoplasty

	**Central**					**Transitional**				
	CG		TG		p*	CG		TG		p*
Criterias	N	%	N	%		N	%	N	%	
Normal histological architecture	0/5	0%	5/5	100%	<0,01	5/5	100%	5/5	100%	1,00
Complete urothelial coating	0/4	0%	3/5	60%	0,44	0/5	0%	4/5	80%	0,05
Urothelial hyperplasia	3/4	75%	5/5	100%	0,44	5/5	100%	0/5	0%	<0,01
Brunn cvsts	2/4	50%	5/5	100%	0,17	0/5	0%	0/5	0%	1,00
Typical extratification of the muscular	1/3	33%	5/5	100%	0,29	1/5	20%	5/5	100%	0,05
Tunic										
Acute inflammation	2/5	40%	3/5	60%	1,00	2/5	40%	3/5	60%	1,00
Chronic inflammation	5/5	100%	5/5	100%	1,00	5/5	100%	5/5	100%	1,00
SIS remnants	3/5	60%	2/5	40%	1,00	2/5	40%	1/5	20	1,00

*Fisher’s exact test.

**Table 2:** Amount of muscle tissue in the central and transitional samples of the animals from the Control and Treated groups

**Control**			**Treated**		
**Region**	**N**	**Average**	**N**	**Average**	**P value**
**Central**	5/5	0.30 ± 0.20	5/5	0.56 ± 0.07	0.04
**Transitional**	5/5	0.54 ± 0.04	5/5	0.68 ± 0.07	0.07

**Table 3:** Amount of collagenous tissue in the central and transitional samples of the animal from the Control and Treat groups

**Control**			**Treated**		
**Region**	**N**	**Average**	**N**	**Average**	**P value**
**Central**	5/5	0.70 ± 0.20	5/5	0.44 ± 0.07	0.04
**Transitional**	5/5	0.46 ± 0.04	5/5	0.32 ± 0.07	0.07

### Clinical, laboratorial and abdominal ultrasonographic evaluations

The clinical evaluation of four animals from the CG revealed pollakiuria, with one animal presenting this alteration in two distinct episodes. The remission of this sign was verified between M3 and M30. Three animals from the TG presented pollakiuria at M1 with subsequent return of the normal urinary frequency at M3 (p=1.00). In addition, four animals from the CG and five from the TG presented macroscopic hematuria at M1 with remission of this sign until M7 (p=1.00). Every other clinical parameter was normal in both groups.

The complete blood count of the animals from the CG and TG revealed no quantitative or morphological variations of the erythrocyte and platelet series. Regarding the leukocyte, intense eosinophilia was verified at M60 in three animals from the CG (average 2.00×10^3^ eosinophil/uL and variation of 1.24×10^3^ a 3×10^3^ eosinophil/uL) and in one animal from the TG (2,324×10^3^ eosinophil/uL) (p=0.52).

The biochemical exams revealed no alterations during the evaluation period, except for the increase of the urea in one animal from the CG (67mg/dL) at M60.

The urine type I exam revealed proteinury in all the animals from the CG and in three animals from the TG during the evaluation period, with one animal from each group presenting this alteration at more than one moment. The presence of magnesium ammonium phosphate crystals in the urinary sediment was verified in three animals from both the CG and TG, this alteration presenting more than once in one animal of each group. With regard to erythrocyte counting, all the animals from the CG presented an amount equal or lower to 5 erythrocytes/uL, except for one animal with 45–50 erythrocytes/uL at M30. Among the animals from the GT, microscopic hematuria was observed at M7 in three dogs (p=0.52), being 10–15 erythrocytes/uL for the first one, 20–15 erythrocytes/uL for the second one, and 75–100 erythrocytes for the third one. Differing to what was found in the animal from the GC, in all the dogs from the TG the great amount of erythrocytes in urine was not followed by leukocyturia and/or bacteriuria. The amount of bacteria was measurable in animals 3 and 5 from the CG, respectively at M7 and M30. In all the cases, *Staphylococcus β hemolytic* was isolated from the urine exam type II*.* No bacteria were isolated or visualized in the exams of the TG animals (p=0.44).

The ultrasonographic exam at M30 showed urinary sediment in four animals from the CG. This, however, was not observed in any animals from the TG (p=0.05). At M60, irregularities were visualized in the area where the SIS was implanted in four animals from the CG, and in two animals from the TG. In association to these irregularities, on three animals from the CG, hyperecoic structures of various sizes forming acoustic shadow were observed, suggesting the presence of uroliths adhered to the SIS. These structures were not visualized in the animals from the TG (p=0.17).

## Discussion

The SIS stands out among several types of grafts due to its capacity to repair the bladder and other urinary tract tissues [8]. The neobladder formed from the SIS, however, differs histologically from the normal bladder as regards the organization and amount of soft muscle fibers, disposed in a disorganized way and in a lower quantity in the SIS neobladder. Besides that, the bladder would become permanently contracted if repaired only with biomaterials, because the bladder’s capacity to contract and relax depends on the normal muscle layer [1,10].

Because of this, HSMC obtained from cadaveric tissues can be an alternative to improving the quality of the repair tissue, since HSMC have the same characteristics of the autologous cells and a reduced immunogenic potential when expanded *in vitro*[11-13]. In addition, although the autologous cells still the first option on cell therapies, the amount of cells obtained is very limited, especially if we take into consideration the age of the patient [13]. Another disadvantage is the possibility of collecting and cultivating neoplastic cells [8,14].

The standardization of the cell culture in this work was essential for the results obtained, amongst which the satisfactory macroscopic reparation of the bladder. However, in spite of the good quality of the reparation tissue at the outer face of the bladder, the intraluminal face of the animals from the CG was characterized, sometimes, by the presence of opaque, irregular and eroded tissue, with the presence of uroliths adhered to its surface. The inferior quality of the reparation tissue on the animals from the CG, comparing to the TG, may be related to the naked lay of the SIS that may predispose to the precipitation and aggregation of the dissolved urine salts. The presence of these salts by itself would result in an increased inflammatory process and formation of uroliths [2]. The inflammatory process would result also in a higher influx of fibroblasts and deposition of collagenous tissue, consequently forming lower quality reparation tissue with wide fibrosis areas [15].

Such findings match the histopathological alterations, characterized by the higher amount of urothelial coating failures in the samples from the CG animals when compared to the TG. This may result from the fact that the muscle cells are important for the maintenance of the structural integrity and organization of the cell graft [2,16]. Grafts that are not seeded with muscle cells present epithelization failures, because although the epithelium has a high regenerative capacity, the muscle cells by yet unknown mechanisms modulate cell proliferation [2]. Perhaps the production of cytokines and other specific metabolites by the muscle cells can provide answers to this issue, but additional studies will be needed.

Additionally, the insufficient revascularization of the graft and the acute inflammation could also have contributed to the unsatisfying results of the histopathological evaluation of the animals from the CG. As mentioned above, the inflammatory cells are responsible for liberating cytokines and other bioactive molecules that could lead to a higher collagen fiber deposition, hindering the restocking of the graft by the muscle cells [17,18].

Thus, the cell coating would waterproof the graft, limiting the toxic effects of the urine on it and, consequently, decrease the inflammatory response [2,14]. According to Zhang *et al*. (2005), such protective function is assigned only to the implanted cells over the intraluminal face of the graft. In this work, however, the HSMC have been seeded over the extraluminal face, which could help to the hypothesis that the muscle cells would participate in the organization and migration of other cells like the urothelial ones.

A cycle is, therefore, created, wherein the muscle cells seeded above the SIS contribute to the epithelization process which, in turn, contributes to muscle fibers deposition in the graft.

The absence of adequate urothelial coating would also justify the main alterations verified on the clinical, laboratorial and abdominal ultrasonographic evaluations, such as the presence of cystitis and urolithiasis. As mentioned previously, the presence of crystals and cell debris, dissolved in a higher quantity in the urine due to the surgical trauma, allied to the anatomical conformation of the female urethra, could result on the crust of the SIS, which would therefore result in an infection and urolithiasis [2,19]. The HSMC would form a protective barrier avoiding the crystals from dissolving in the urine precipitates among the collagen fibers of the graft, consequently contributing to the absence of cystitis and urolithiasis in the animals from the TG [2,20].

Besides that, the higher amount of collagen fibers in the acellular SIS would result in a lower capacity of bladder contractibility, thus resulting in a higher quantity of residual urine, which could also have predisposed an infection of the urinary tract in the animals from the CG [21].

## Conclusion

The SIS seeded with HSMC, when compared to the acellular graft, resulted in a typical histological architecture in the region of the cystoplasty, characterized by the presence of better individualized and organized tunics; and promotes the tissue repair process with higher percentage of smooth muscle fibers over the collagen fibers.

The acellular or seeded SIS does not induce clinical, laboratorial or histological alterations compatible to rejection.

## Competing interests

The authors declare that they have no competing interests.

## Authors’ contributions

VJVR has made substantial contributions to the conception of the study and acquisition of data; FG carried out the histopathological and immunocytochemistry studies; NSR carried out the histopathological studies and contributed to the interpretation of data; HAM carried out the quantitative analysis of the muscle and collagen fibers and performed the statistical analysis; LSLSM has been involved in drafting the article and carried out the cellular culture process; and CVSB conceived the study and participated in its design and coordination, and helped to draft the article. All authors read and approved the final article.
